# Prevalence and determinants of anemia among women of reproductive age in Thatta Pakistan: Findings from a cross-sectional study

**DOI:** 10.1371/journal.pone.0239320

**Published:** 2020-09-24

**Authors:** Sumera Aziz Ali, Zahid Abbasi, Babar Shahid, Ghazal Moin, K. Michael Hambidge, Nancy F. Krebs, Jamie E. Westcott, Elizabeth M. McClure, Robert L. Goldenberg, Sarah Saleem

**Affiliations:** 1 Department of Epidemiology, Mailman School of Public Health, Columbia University, New York City, New York, United States of America; 2 Department of Community Health Sciences, Aga Khan University, Karachi, Pakistan; 3 Department of Pediatrics, Section of Nutrition, University of Colorado Anschutz Medical Campus, Aurora, Colorado, United States of America; 4 Regional Triangulate Institute International, Research Triangle Park, North Carolina, United States of America; 5 Department of Obstetrics and Gynecology, Columbia University, New York City, New York, United States of America; University of Zurich, SWITZERLAND

## Abstract

**Background:**

Anemia is a major public health concern among women of reproductive age leading to high maternal mortality in low- and middle-income countries. Of the prior studies conducted in Pakistan, most focused on large urban areas and did not explore the determinants of anemia among women of reproductive age (WRA) across socio-demographic, dietary, reproductive, and biological domains. Thus, we aimed to study the prevalence and determinants of anemia among WRA in rural Pakistan.

**Methods:**

We conducted a cross-sectional study in the Thatta district of Pakistan from September 2018 to January 2019 and enrolled 150 non-pregnant, married women. Data collectors administered a structured questionnaire to collect sociodemographic, reproductive and dietary data from women, who also provided stool and blood samples. We classified all WRA as anemic if their hemoglobin was <12.0 g/dl. We performed logistic regression analysis to calculate adjusted odds ratios (aOR) and their respective 95% CIs to assess the determinants of anemia.

**Results:**

In our study, 61.3% of the enrolled women were anemic. In the multivariable analysis, we found that factors such as serum iron levels of less than 50 μg/dl (aOR: 7.17; 95% CI (2.94, 17.47)), history of breastfeeding (aOR: 2.43; 95% CI (1.04, 5.72)), living in a katcha house (aOR: 6.61; 95% CI (2.21, 19.87)), no consumption of meat (aOR: 4.18; 95% CI (1.66, 9.96)) were significantly associated with anemia among WRA. A history of more than one abortion (aOR: 0.06; 95% CI (0.01, 0.33) appeared protective for its association with anemia.

**Conclusion:**

Our findings demonstrate a high burden of anemia and its complex determinants among WRA in rural Pakistan. A combination of nutritional and educational strategies should be designed to encourage rural women to consume iron-rich foods in their diet with an access to adequate food. Breastfeeding women should be encouraged to consume extra calories with sufficient intake of the food to continue exclusive breastfeeding and reserve the iron stores through amenorrhea to prevent themselves from becoming anemic.

## Introduction

Anemia is one of the major public health issues among women of reproductive age (WRA), as it leads to high maternal morbidity and mortality and adverse pregnancy outcomes [1]. Anemia occurs when hemoglobin (Hb) concentration falls below an established cut-off value (<12.0 g/dl) among WRA, consequently impairing the capacity of the blood to transport oxygen to the body [2–4]. About half a billion of WRA are anemic worldwide, with a higher burden of anemia in low and middle-income countries (LMICs), affecting nearly two-thirds of WRA [5, 6]. More specifically, 41.9% of WRA are anemic in South-East Asia [6]. This is in contrast to the burden of anemia in developed countries, such as in Europe where 2–5% of WRA are classified as anemic [7]. Like other LMICs, there is a high burden of anemia in Pakistan [8]. Studies have shown that anemia affects 41.7% to 77.0% of WRA in Pakistan [8, 9]. Anemia is more prevalent in the rural areas of Pakistan, where it is often severe and linked to adverse health consequences such as postpartum hemorrhage, preterm delivery or stillbirth and low birth weight babies [10]. Furthermore, a study conducted by Hambidge et al 2019 found that anemia is an important and potentially modifiable risk factor on birth outcomes among rural Pakistani women [11]. More specifically, this study demonstrated that maternal anemia at baseline modified the treatment effect for birth outcomes such as birth weight, weight to length ratio as the improvements in these outcomes were significantly greater for Arm 1 (commencing nutrition intervention prior to conception) vs. Arm 2 (commencing same nutrition intervention early in gestation) [11].

Although few studies have investigated the determinants of anemia in Pakistan, certain gaps need to be addressed [12–14]. First, existing studies mainly focused on large urban areas of Pakistan rather than rural areas where the burden of anemia is higher. In addition, there are differences in the socio-demographic, cultural, economic, and dietary factors between rural and urban areas, which do not accurately characterize anemia in a rural setting [15]. Moreover, rural areas have limited sources of income with poor access to health facilities and other amenities such as electricity, modes of communication and access to health care facilities [16, 17]. Therefore, studies of urban populations may not be relevant in the rural context of Pakistan. Second, anemia is a complex and multifactorial phenomenon that should be understood holistically. Current studies in urban settings provide important insights about sociodemographic and reproductive determinants, but leave key biological and dietary determinants unaddressed and therefore, poorly understood [18–20]. Lastly, investigators have not utilized objective measurements of biological factors such as iron, ferritin, B12, and folic acid in the serum, hookworm in the stool, and a malarial parasite in the blood. Rather, their results are often based on self-reported data, which are subjective and may have produced biased estimates of associations between biological determinants and anemia among WRA.

There is a need to investigate the underlying determinants of anemia comprehensively across the range of socio-demographic, reproductive, dietary, and biological domains in rural Pakistan, which have not been previously studied in this context. Understanding these determinants holistically will enable the development of local strategies and targeted interventions to address the burden of anemia among WRA in rural Pakistan. Hence, the overarching objectives of this study are to measure the prevalence and determinants of anemia among WRA in rural Pakistan. We hypothesized that poor, less educated, older, and multiparous women who consume iron-deficient diet with abnormal biomarkers will be more anemic than younger, nulliparous, well-privileged women who consume iron-rich diet.

## Materials and methods

### Study population and sample

We conducted a cross-sectional study in the district of Thatta, Pakistan from September 2018 to January 2019. We examined the associations of sociodemographic, reproductive, dietary, and biological determinants of anemia in 150 WRA participating in the Women First (WF) preconception maternal nutrition trial [21]. At the time of enrollment in the primary WF trial, women were eligible if they had 0–5 children and planned to conceive in the future after being enrolled in the WF primary study. We used a subset of women from the WF database (385 women) who provided biological samples in the primary trial, who had conceived during their enrollment in the WF primary study, and had delivered their baby [21]. For this cross-sectional study, we randomly selected study participants using the WF database as a sampling frame. Eligibility was contingent on being a resident of the Thatta district, not being pregnant at the time of survey, agreeing to participate, and intending to provide biological samples after enrollment in the cross-sectional study.

### Data collection tool (questionnaire)

We used a structured and validated questionnaire to collect data on socio-demographic, reproductive, anthropometric, dietary, and biological determinants from WRA. We identified possible determinants of anemia based on the literature pertaining to the risk of development of anemia among women in LMICs [19]. To develop a questionnaire for the proposed cross-sectional study, we chose a series of validated questions from different studies that have been conducted in Thatta Pakistan. For example, the Pakistan Demographic Health Survey 2012–2013 (PDHS-2012-2013) was one of such sources to choose the relevant validated questions [15]. We selected questions related to socio-demographic characteristics from the PDHS-2012-2013 survey and adapted the questions wherever necessary [15]. Secondly, a maternal and newborn health registry (MNHR) is placed in Thatta district [20], whereby data is being collected on the socio- demographic, and reproductive characteristics of WRA in a similar setting and same population, where we undertook the cross-sectional study [18]. The MNHR has validated the questions in a similar population that also helped me to use several relevant questions for the current cross-sectional study [18]. Lastly, an individual randomized nutritional trial (Woman First) was conducted from 2012 to 2018 in Thatta district where MNHR is based [19, 21]. The trial used 20 validated questionnaires to explore socio-demographic, reproductive, dietary factors, anthropometric measurements, and biological factors among WRA in the same population [19]. Since all these questionnaires were relevant to the proposed study; therefore, we choose the most appropriate questions from these validated questionnaires and adapted these wherever required to develop and finalize the questionnaire for the cross-sectional study. After developing and finalizing the questionnaire from three main sources, we translated the questionnaire into the local language of district Thatta (Sindhi) followed by back-translation. To check the flow of questions, we pre-tested questionnaire on the WRA other than the study setting of Thatta but having similar characteristics to the study sample. The questionnaire consisted of different sections and collected socio-demographic, reproductive, dietary, and anthropometric data.

### Study procedures & data collection

After assessing the eligibility criteria and receiving written informed consent, trained study staff, who were familiar with the local language (Sindhi), administered a structured and validated questionnaire to the enrolled women in their households. The study staff asked women about various socio-demographic, reproductive and dietary measures including maternal age, parity, education, working status, water supply, sanitation, age at the time of marriage, history of any previous adverse pregnancy outcome, date of the last delivery, consumption of fruits, vegetables and meat in their diet, and use of smokeless tobacco. The study staff also measured weight (in kilograms), height (in meters), waist circumference (in centimeters (cm)), hip circumference (cm), and mid-upper arm circumference (cm). Using a digital weighing scale, the study staff measured the weight of WRA while barefoot and wearing light clothes to the nearest 0.1 kg. The same staff measured the height of a woman barefoot to the nearest of 0.1 cm using a stadiometer. Before the measurement, the staff calibrated scales to zero. The staff measured mid-upper arm circumference on the upper right arm at the midpoint of the acromion process and the tip of the olecranon (precision 1mm). Similarly, the research staff measured waist circumference just above the iliac crest in the horizontal plane, and hip circumference at the point yielding the maximum circumference over the buttocks, all using a standard measuring tape to the nearest 1mm. We calculated body mass index (BMI) as a ratio of weight (in kilograms) to the square of height (in meters) and the study staff recorded BMI as a continuous variable. We categorized BMI as underweight (less than 18.5 kg/m2), normal (18.5–24.9 kg/m2), overweight (25.0–29.9 kg/m2), and obese (≥30.0 kg/m2) [22]. For regression analysis, we merged overweight and obese categories into a single category as overweight/obese due to low numbers in the obese category.

A trained and professional phlebotomist collected biological samples including blood and stool samples from the enrolled women. The phlebotomist collected around 12–15 mL of venous blood by following the standard procedures [19]. The phlebotomist transferred all biological samples from Thatta collection points to the main laboratory of the Aga Khan University Hospital in Karachi, Pakistan by maintaining the cold chain. The trained laboratory staff and microbiologist processed the blood samples to measure complete blood count (CBC), serum iron and ferritin levels, serum B-12 and folic acid levels. In addition, they used the blood samples to measure the inflammatory markers including erythrocyte sedimentation rate (ESR) by the Westergren method, and C-Reactive protein (CRP) using a high sensitivity ELISA assay [23, 24]. Moreover, they used an immunochromatographic test (ICT) technique to identify the malarial parasite in the blood and examined blood for hemoglobinopathies including thalassemia minor and major by hemoglobin (Hb) electrophoresis test [25, 26]. Furthermore, they used stool samples to examine different worms in the stool to primarily assess hookworm infestation. They identified parasites and eggs in the stool samples, using a microscope with conventional techniques such as direct wet smear stain (Lugol stain) [27].

### Primary outcome

The primary outcome of this study was anemia and we used hemoglobin levels (grams per deciliter) in the blood to classify a woman as anemic. More specifically, we used the World Health Organization (WHO) cut-off of a hemoglobin level <12.0 g/dL to classify a non-pregnant woman aged ≥15 years as anemic [28–32]. For the current analysis, we created a binary variable of anemia and no-anemia.

### Potential determinants of anemia

Independent variables in this study consisted of various socio-demographic, reproductive, dietary, and biological determinants. The socio-demographic determinants included woman’s age (in years), literacy status (illiterate / literate), residence (urban/ rural), type of house (pucca house: made of concrete, plastered walls, and roof / katcha house: made of wood, clay, and stalks), religion (Hinduism/Islam), current working status (are you working currently to earn money: Yes/No), having electricity (Yes/No), type of family system (nuclear/joint family).

Reproductive determinants included age at the time of marriage (in years), gravidity (total number of pregnancies), parity (number of children born after 20 weeks of gestation), history of adverse pregnancy outcomes including stillbirths and abortions (Yes/No), breastfeeding status at the time of the survey (Yes/No), current and ever use of family planning method (Yes/No), and menstrual cycle (regular/irregular).

Dietary determinants included intake of fruits and vegetables in past one month (Yes / No), consumption of meat (Yes/ No), and history of chewing smokeless tobacco (Yes/No). In addition, we measured biological determinants such as serum iron (normal range: 50–170 μg/dl), serum ferritin (normal range: 10 to 150 ng/ml), serum folic acid (normal range: ≥ 2.5–20 ng/ml), serum CRP (normal range: 0–0.5mg/dl), ESR (normal range: 0–20 mm/ 1st hr), thalassemia minor or major (yes/no), malarial parasite (positive/negative), and parasites in the stool (seen/unseen).

### Statistical methods

To describe the characteristics of the study population, we reported frequencies and proportions. We used a Chi-squared test or Fisher’s exact test to assess the frequency distribution and the relationship between determinants and anemia status. Similarly, we performed bivariate logistic regression analyses to determine the individual effect of each significant determinant on anemia status before the multivariable logistic regression analysis. We used multicollinearity diagnostics such as variance inflation factor (VIF) and tolerance (reciprocal of VIF) to assess multicollinearity between independent variables [33]. Any VIFs of more than 4 were investigated, while VIFs greater than 10 or tolerance less than 0.1 indicated serious multicollinearity requiring correction by dropping one of the variables [33, 34].

Finally, we conducted a multivariable logistic regression analysis to determine the effect of each determinant on anemia by including all predictors and covariates in a single multivariable model using a purposeful selection method [35]. We used a p-value cut-off of <0.05 to determine if a variable should be included in the final regression model. We presented the results of regression analyses with crude/unadjusted odds ratios (OR) and adjusted odds ratios (aOR) with 95% confidence intervals (CIs). We used SPSS 19.0 to analyze the data.

### Ethical considerations

Ethical approval for this study was obtained from the Aga Khan University Ethical Review Committee (2018–0262–342). All study participants provided written informed consent. Voluntary participation and the right to ask any questions and decline participation at any time were emphasized during data collection.

## Results

### Characteristics of women of reproductive age

We found that more than a quarter of the women (28.0%) were younger than 25 years old and 40.0% were 25–29 years old as shown in Table 1. Moreover, 82.0% of the women were not able to read or write, a similar proportion of the women (81.3%) were residents of rural areas, and 44.7% reported to be living in a katcha house. More than half of the study participants (55.3%) did not report working to earn money, and around three-fourths (73.3%) of them were living in a joint family system (Table 1). Around three-fourths of the study participants (71.3%) had given birth to at least three children, and 8.7% had a history of more than one abortion. Slightly more than a third of women (36.0%) reported not breastfeeding their children at the time of the study, and 46.0% had a normal BMI of 18.5-25kg/m^2^. Regarding the women’s diet, we found that 41.3% of the women had never consumed fruits or vegetables in the past month, and 68.7% had never eaten meat in the past month (Table 1).

**Table 1:** Socio-demographic, reproductive and dietary determinants of women of reproductive age by anemia status in Thatta Pakistan, crude odds ratios and 95% CI (n = 150).

Characteristics	Total n (%)	Anemia n (%)	No Anemia n (%)	Odds Ratio	95%CI	P-value*
**Maternal age (Years)**						
< 25	42 (28.0)	24(26.1)	18(31.0)	1		
25–29	60(40.0)	36 (39.1)	24(41.4)	1.13	0.51–2.50	0.63
≥30	48 (32.0)	32(34.8)	16(27.6)	1.50	0.64–3.53	
**Parity**						
≤2	43 (28.7)	24(26.1)	19(32.8)	1		
3–4	66 (44.0)	39 (42.4)	27(46.6)	1.14	0.53–2.48	
>4	41 (27.3)	29(31.5)	12(20.7)	1.91	0.77–4.71	0.33
**Educational Status of women**						
Literate	27 (18.0)	18(19.5)	09(15.5)	1		
Illiterate	123 (82.0)	74(80.4)	49 (84.4)	0.75	0.28–1.76	0.45
**Current working status of woman**						
No	83 (55.3)	54(58.7)	29(50.0)	1		
Yes	67 (44.7)	38(41.3)	29(50.0)	0.70	0.36–1.36	0.29
**Place of residence**						
Urban	28 (18.7)	18(19.6)	10(17.2)	1		
Rural	122 (81.3)	74(80.4)	48(82.8)	0.85	0.36–2.01	0.72
**Type of Family System**						
Nuclear	40 (26.7)	23(25.0)	17(29.3)	1	0.59–2.59	0.56
Joint	110 (73.3)	69(75.0)	41(70.7)	1.24		
**Type of House**						
Pakka	83 (55.3)	44(47.8)	39(67.2)	1		
Katcha	67 (44.7)	48(52.2)	19(32.8)	2.24	1.13–4.44	0.02
**Age at the time of marriage**						
>18	79 (52.7)	42(45.7)	29(50.0)	1		
≤ 18	71 (47.3)	50(54.3)	29(50.0)	1.19	0.62–2. 29	0.60
**Number of abortions**						
≤ 1	137 (91.3)	89(96.7)	48(82.8)	1		
>1	13 (8.7)	3(3.3)	10(17.2)	0.16	0.04–0.62	0.005
**Currently Breastfeeding**						
No	54 (36.0)	29(31.5)	25(43.1)	1		
Yes	96 (64.0)	63(68.5)	33(56.9)	1.65	0.83–3.25	0.15
**Current usage of contraception**						
Yes	45 (30.0)	29(31.5)	16(27.6)	1		
No	105 (70.0)	63(68.5)	42(72.4)	0.81	0.40–1.71	0.61
**Intake of vegetables (past one month)**						
Yes	88 (58.7)	51(55.4)	37(63.8)	1		
No	62 (41.3)	41(44.6)	21(36.2)	1.42	0.72–2.78	0.31
**Intake of fruits (past one month)**						
Yes	88 (58.7)	52(56.5)	36(62.1)	1		
No	62 (41.3)	40(43.5)	22(37.9)	1.26	0.64–2.46	0.50
**Intake of meat (past one month)**						
Yes	47 (31.3)	22(23.9)	25(43.1)	1		
No	103 (68.7)	70(76.1)	33(56.9)	2.41	1.18–4.88	0.01
**Chew smokeless tobacco**						
No	48 (32.0)	33(35.9)	15(25.9)	1		0.20
Yes	102 (68.0)	59(64.1)	43(74.1)	0.62	0.30–1.28	
**BMI (kg / *m***^**2**^**)**						
Overweight & Obese	12 (8.0)	3 (3.3)	9 (15.5)	1		
Underweight	69 (46.0)	43(46.7)	23(39.7)	4.96	1.23–20.01	0.04
Normal	69 (46.0)	46(50.0)	26(44.8)	6.00	1.48–24.31	

*P-values have been calculated using Chi-squared test.

We found that 61.3% of women were anemic in the study, and a similar proportion of the women (62.0%) had low serum iron levels (Table 2). We found that 42.7% of the women had lower serum ferritin values; however, only 6.0% of the women had low folic acid levels relative to the normal range of > 2.6ng/ml (Table 2). All of the women had serum B12 levels within the normal range (>150 pg/ml). Around 10.0% of the women had higher values of CRP than the normal range of 0–0.5mg/dl, and around 50.0% of the women had higher values for ESR than the normal range of 0–20 mm/ 1st hr. We found that only 8.7% of the women had any kind of underlying hemoglobinopathy, and 16.0% of the women’s bloods were positive for the malarial parasite and 15.2% of the women had parasites in their stool as shown in Table 2.

**Table 2:** Biological determinants of women of reproductive age by anemia status in Thatta Pakistan, crude odds ratios and 95%CI (n = 150).

Characteristics	Total n (%)	Anemia n (%)	No Anemia n (%)	Odds Ratio	95%CI	P-value*
**Serum Iron (μg/dl)**						
≥ 50	57 (38.0)	23(25.0)	34 (58.6)	1		
< 50	93 (62.0)	69(75.0)	24(41.4)	4.25	2.1–8.59	<0.001
**Serum Ferritin (ng/ml)**						
> 10	86 (57.3)	45(48.9)	41 (70.7)	1		
≤ 10	64 (42.7)	47(51.1)	17(29.3)	2.52	1.25–5.06	0.01
**Serum Folic Acid (ng/ml)**						
> 2.6	141 (94.0)	89 (96.7)	52 (89.7)	1		
≤ 2.6	9 (6.0)	3(3.3)	6(10.3)	0.29	0.07–1.22	0.09
**Serum C-Reactive Protein (mg/dl)**						
0–0.5	102 (89.5)	47(88.7)	32(82.1)	1		
> 0.5	12 (10.5)	6(11.3)	7(17.9)	0.58	0.18–1.89	0.36
**ESR (mm/ 1**^**st**^ **hr)**						
0–20	60 (50.4)	34(47.9)	26(54.2)	1		
> 20	59 (49.6)	37(52.1)	22(45.8)	1.29	0.62–2.68	0.50
**Hemoglobinopathies on Hb electrophoresis**						
No	137 (91.3)	83 (90.2)	54(93.1)	1		
Yes	13 (8.7)	9 (9.8)	4(6.9)	1.46	0.43–4.99	0.54
**Malarial parasite**						
Negative	126 (84.0)	78(84.8)	48(82.8)	1		
Positive	24 (16.0)	14(15.2)	10 (17.2)	0.86	0.35–2.09	0.74
**Parasites in stool**						
Not seen	125 (83.3)	78 (84.8)	47(81.0)	1		
seen	25 (16.7)	14(15.2)	11(19.0)	0.76	0.32–1.83	0.55

*P-values have been calculated using Chi-squared test.

### Sociodemographic, reproductive, and dietary characteristics of women of reproductive age by anemia status

We found no differences between anemic and non-anemic women by age, educational level, parity, working status, place of residence, and type of family system (Table 1). However, we found that a significantly higher proportion of anemic women (52.5%) were living in a katcha house when compared with 32.8% of non-anemic women (p-value: 0.02). Similarly, 68.5% of anemic and 56.9% of non-anemic women reported breastfeeding their children. A significantly higher proportion of anemic women (76.1%) had never consumed meat in the past month compared with non-anemic women (56.9%; p-value: 0.01). About half of anemic women (46.7%) were underweight, while 39.7% of the non-anemic women were underweight (Table 1).

The study also found that three-fourths of the anemic women (75.0%) had lower serum iron levels compared to 41.4% of the non-anemic women. Similarly, around half of anemic women (51.1%) had lower serum ferritin levels when compared to 29.3% of non-anemic women as shown in Table 2. There were no differences between anemic and non-anemic women by levels of serum folic acid, CRP, ESR, hemoglobinopathies, and malarial parasite in the blood and stool parasites (Table 2).

### Determinants of anemia among women of reproductive age: Findings from a bivariate analysis

We assessed and found multiple statistically significant associations between covariates and anemia in bivariate analyses. More specifically, we assessed the association between various sociodemographic, reproductive, dietary, and biological factors and anemia among WRA. We found that women with more than four children had a 1.91(OR = 1.91; [95% CI: 0.77, 4.71]) times the odds of being anemic when compared with women having less than 2 children (Table 1). Meanwhile, women living in a katcha house had a 2.24 (OR = 2.24; [95% CI:1.13, 4.44]) times the odds of being anemic when compared with their counterparts. While exploring the association between reproductive factors and anemia, we found that women who were breastfeeding their children at the time of the study had a 1.65 (OR = 1.65; [95% CI: 0.83, 3.25]) times the odds of being anemic when compared with their counterparts; however, results were not statistically significant. Furthermore, women with low and normal BMI had 4.96 (OR = 4.96; [95% CI: 1.23, 20.01]) and 6.00 (OR = 6.00; [95% CI: 1.48, 24.31]) times the odds of being anemic, respectively, when compared with overweight and obese women. We also assessed the association between women’s diet and anemia in the bivariate analysis. We found that women who never consumed fruits and vegetables in the last month had 1.26 (OR = 1.26; [95% CI: 0.64, 2.46]) and 1.42 (OR = 1.42; [95% CI: 0.72, 2.78]) times the odds of being anemic when compared to women who consumed fruits and vegetables, respectively; however, these results were statistically insignificant (Table 1). Women who never consumed meat in the last month had 2.41 times the odds of being anemic when compared women who reported consuming meat in the past one month (OR = 2.41; [95% CI:1.18, 4.88]). Regarding the association between biological factors such as serum iron levels and anemia, we found that women with low serum iron levels had 4.25 (OR = 4.25; [95% CI:2.1,8.59]) times the odds of being anemic when compared to women having normal serum iron levels as shown in Table 2. In contrast to other potential factors, we found that a history of more than one abortion was found to be protective for its association with anemia (OR = 0.16; [95% CI:0.04, 0.62]).

### Determinants of anemia among women of reproductive age: Findings of multivariable analysis

**Fig 1:**
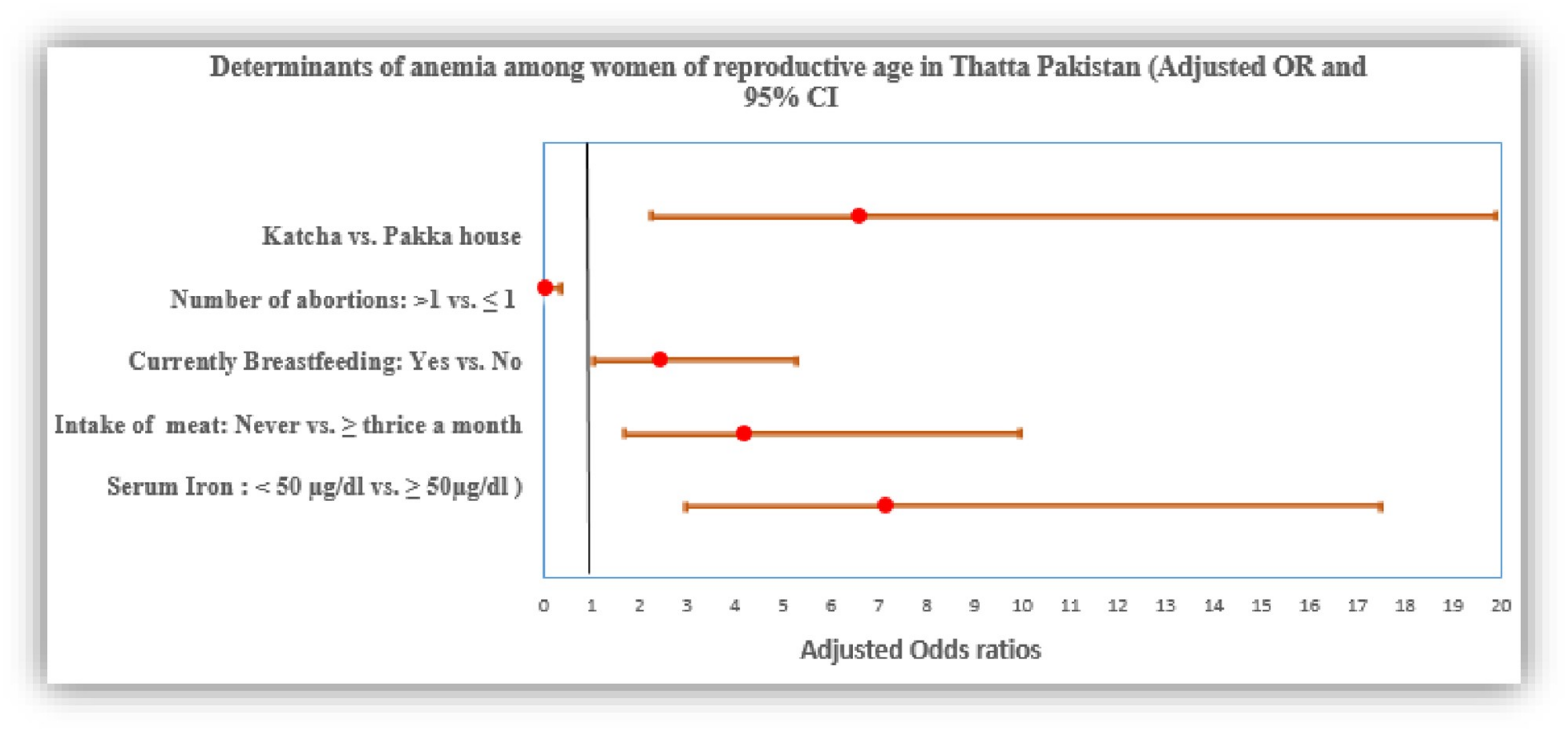
Determinants of anemia among women of reproductive age in Thatta Pakistan [adjusted OR and 95% CI].

The results of multivariable analyses demonstrated that low serum iron level, living in katcha house, no consumption of meat in the past month, and history of breastfeeding increased the risk of anemia (Fig 1). More specifically, significant and positive associations between low serum iron levels (aOR = 7.17; 95% CI [2.94, 17.47]), living in a katcha house (aOR = 6.61; [95% CI: 2.21, 19.87]), no consumption of meat in the past month (aOR = 4.18; [95% CI [1.66, 9.96]) and anemia persisted in the multivariable analysis. Moreover, the association between history of breastfeeding and anemia turned out to be statistically significant in the adjusted model (aOR = 2.43; [95% CI:1.04, 5.72]). A history of more than one abortion (aOR: 0.06; 95% CI (0.01, 0.33) appeared protective for its association with anemia as shown in Fig 1.

## Discussion

This was one of the first studies in Pakistan that measured the burden of anemia and its determinants comprehensively across socio-demographic, reproductive, dietary, and biological domains among WRA in rural settings. Our study demonstrated a high prevalence of anemia among WRA in a rural area of Pakistan. Factors such as low serum iron levels, no consumption of meat, living in a katcha house, and history of breastfeeding were found to be risk factors for anemia among WRA. However, women with more than one abortion were found to be at lower risk for anemia. We did not find any differences between anemic and non-anemic women by malarial parasite in the blood and stool parasites. Thus, presence of a malarial parasite or stool parasite did not turn out to be important factors related to anemia in this study. The results on the prevalence and determinants of anemia among Pakistani women should be interpreted in their economic and socio-cultural context.

Our observation regarding the prevalence of anemia is consistent with other studies conducted in different rural areas of Pakistan, where the prevalence of anemia has been found to be between 41.7% to 77.0% [8, 9, 36, 37]. However, it is important to note that a direct comparison of our findings with earlier studies conducted in Pakistan may not be possible because of the differences in the number and composition of the study subjects enrolled, study settings and socio-cultural circumstances.

The association of low serum iron levels with a higher risk of anemia in this study is compatible with the other studies conducted in urban areas of Pakistan and other low-income countries, such as Vietnam and Ethiopia [31, 38–40]. Although studies conducted in urban areas of Pakistan or Ethiopia did not measure serum iron levels in the blood, their results demonstrate that women not consuming iron-rich foods or iron supplements and those with food insecurity are at higher risk of anemia than women who consume iron-rich foods or take iron supplements [31, 38–40]. The consistently positive association between low serum iron levels and anemia may be due to poor diet quality and poor intake of iron-rich foods in our study population. This was further evident from our study’s findings where two-fifths of the women reported no consumption of vegetables and fruits in the prior month and more than two-thirds of the women reported no consumption of meat in the prior month. Thus, inadequate intake of iron, predominantly caused by reduced access to heme iron, which is primarily found in meat and is highly bioavailable, can contribute to iron deficiency [41–43]. Furthermore, Lander et al. [44] reported that four-fifths of rural Pakistani women in the same district (Thatta) were found to consume a diet with inadequate diversity, which might make WRA susceptible to anemia in Thatta district [44]. Dietary intake data from a similar study also indicated rural Pakistani women had the lowest mean intake of calories, protein and key micronutrients [44]. In addition to inadequate dietary diversity, the use of smokeless tobacco, e.g. “gutkha” is common among rural Pakistani women and has been shown to reduce appetite and cause inflammation and thus may have affected the intake of iron-rich foods in our study population [45].

We found a positive association between poor consumption of meat and anemia in our study population and this finding is consistent with other studies, which reported that poor intake of meat is a risk factor of anemia among WRA [46, 47]. In rural Vietnam, the consumption of meat of at least three times a week is more common in non-anemic women than in anemic women [48]. Likewise, anemic women in one of the observational studies reported lower intake of red meat than non-anemic women [40]. This finding can be explained by the fact that poor intake of meat can lead to low levels of iron in the body, thus resulting in anemia. Overall, two types of dietary iron have been found; one is heme iron that comes mainly from animal flesh foods and the other is non-heme iron, which is the only type found in plant-based foods, such as grains and vegetables [49, 50]. In humans, the heme form is the most bioavailable, with an estimated absorption rate of 15%–35% when compared to absorption rate of 2% to 20% for non-heme iron [50, 51].

As expected, women living in a katcha house were more likely to be anemic than their counterparts. Living in a katcha house is one of the proxy indicators of poor socioeconomic status, which is further related to the intake of a poor diet [28]. This relationship between poor socio-economic status and anemia is consistent with other studies conducted in different parts of Asia, Africa, and urban areas of Pakistan [28, 37, 39, 52]. For instance, a study conducted in northern Pakistan revealed that women with a poor socioeconomic status were more likely to be anemic than women with a higher socioeconomic status [37]. Likewise, a study conducted in Bangladesh found that rich women were less likely to be anemic when compared to poor women [28]. Collectively, these findings can be explained by the fact that a poor woman’s purchasing power is meager; therefore, she cannot afford iron-rich food, such as meat and certain iron-rich fruits and vegetables, and is therefore consumes a non-nutritious diet consequently resulting in anemia [53, 54]. Moreover, due to food insecurity, these poor women also tend to consume smokeless tobacco and non-nutritious substances such as fuller’s earth, clay, and ice to reduce their appetite, and consequently, are more susceptible to anemia due to a lack of adequate nutrition [55]. Generally, a woman’s diet also reflects her socioeconomic status. Therefore, living in katcha homes and consuming low meat may all reflect a more marginal living circumstances of a woman, including more risk of poor sanitation and poorer health status (more inflammation as reflected by higher values of inflammatory markers and poorer iron absorption). Thus, diet may well be part of the problem, but it is complicated and interconnected, and it is likely more than just not eating an adequate iron rich diet.

A history of breastfeeding was found to be associated with anemia among WRA, and this finding is consistent with findings from other low-income countries [39, 56]. For example, a study conducted in Ethiopia found that lactating women were at higher risk of anemia when compared with non-lactating women [39]. There is evidence that a lactating woman needs to consume more calories with a more diversified diet than non-lactating women [57]. Studies from Pakistan have reported that women during pregnancy and lactation tend to avoid some foods (due to misconceptions and beliefs) such as beef, eggs, brinjal, fish and citrus fruits as these are considered hot and could have ill effects on their babies [58, 59]. Since the amenorrhea associated with full exclusive breastfeeding is generally assumed a protective factor for iron status; insufficient intake of adequate diet could lead to insufficient milk production and thus shorter exclusive breastfeeding and resumption of menses, which eventually leads to worse iron status and anemia [60].

Contrary to findings reported in the literature [61–63], a history of more than one abortion was found to be protective against anemia in our study; however, this finding should be interpreted with caution. There could be different plausible explanations for this inverse finding. Women with more than one abortion were not able to complete the nine months of pregnancy duration, thus preventing them from physiological anemia caused by pregnancy [64]. This means that these women avoided anemia that might have occurred had they completed their full-term pregnancy, as pregnancies generally result in a reduction in the concentration of red blood cells [65]. Secondly, our study measure regarding the history of abortion was binary without any further details about the abortions. For example, we did not ask about the type of abortion from these women [spontaneous vs induced]. In addition, we did not ask women about how long they had experienced bleeding after abortion, nor did we investigate the volume of blood loss or any clots in the blood during the abortion. This lack of information limits our ability to interpret these results. Lastly, it could be possible that women may not have correctly reported their history of abortion due to the sensitive nature of the issue and stigma associated with abortion, or they might have considered a spontaneous abortion as normal menstrual bleeding. If this reporting were differential across anemic and non-anemic women, it could have led to a higher number of women with less than one abortion among anemic than non-anemic women. Due to these potential limitations for this inverse finding, it is hard to make any conclusion from this protective association between abortion and anemia, and this needs to be explored further with sufficient details about the history of abortion.

### Strengths and limitations

This is one of the first studies in rural Pakistan that investigated potential determinants of anemia comprehensively across sociodemographic, reproductive, dietary and biological domains. In addition, the random sampling of women suggests that these data are representative of the overall population in the district. In addition, unlike other studies conducted in a few urban areas, our data regarding biological determinants of anemia were based on objective measurements rather than relying on self-reported data from women. However, there are some inherent limitations. First, the cross-sectional nature of the survey does not allow temporal precedence between various socio-demographic, dietary, reproductive, and biological determinants, and anemia to be unambiguously determined. However, unlike other cross-sectional studies reported in the literature, our study explored the determinants of anemia holistically across socio-demographic, reproductive, dietary, and biological domains. Secondly, our dietary history questionnaire queried only what was eaten in the prior month and may not have fully represented the daily or seasonal dietary patterns of the women. Although the measured dietary history did not represent daily or seasonal dietary patterns, it did indicate monthly dietary patterns of WRA among rural women. A third limitation is that due to the sensitive nature of some questions such as the use of contraception and the history of abortion, women might not have reported the information accurately. However, we tried to overcome this limitation by asking the sensitive questions in privacy and assured participants about the confidentiality of their responses. Lastly, the small sample size of the study may limit our findings to be generalized to the larger group of rural Pakistani women, thus more longitudinal and robust tepidemiological studies with larger sample size are required in the future.

## Conclusion

Our study confirmed that anemia is a major public health problem in women of reproductive age in rural Pakistan and a large proportion of women were found to have low levels of serum iron. Lack of dietary iron intake mainly contributes to iron deficiency among poor rural Pakistani women. These findings provide a rationale to take necessary steps aimed at alleviating iron deficiency.

There could be several policy implications for this study in the future. The government should target disadvantageous rural areas and social groups where the purchasing power, knowledge, and access to health care facilities are limited. More specifically, government of Pakistan need to develop some mechanisms to reduce the poverty at the household level by creating job opportunities for the people living in remote areas of Pakistan. One of the ways for poverty alleviation is to ensure good education in the schools both for boys and girls mainly in the rural areas, which are highly reliant on public schools. Secondly, government can provide small loans or introduce social safety-net programs to help people living below the poverty line with proper monitoring of the funds allocated for such poor households.

In addition, there is a need to design combined nutrition and educational strategies to encourage rural women to consume iron-rich foods in their diet. Proactive efforts can be made to educate women through effective campaigns for motivating them to take iron tablets according to the prescribed schedule. Moreover, breastfeeding women should be encouraged to consume extra calories during lactation to prevent themselves from becoming anemic. Similarly, ensuring poor women’s access to information on risks of eating an iron-poor diet and having a less adequate diet during lactation is also important to reduce anemia among women in rural Pakistan.

## Supporting information

S1 File (PDF)

S2 File (SAV)
