# Supplementary material for: Prevalence and determinants of anemia among women of reproductive age in Thatta Pakistan: Findings from a cross-sectional study
Source: PLoS One. 2020 Sep 24;15(9):e0239320. doi: 10.1371/journal.pone.0239320 (PMC7514090; doi:10.1371/journal.pone.0239320)
Supplement: S1 File — (PDF) [file pone.0239320.s001.pdf]

Prevalence and determinants of anemia among women of reproductive age in Thatta Pakistan

**SECTION A: SOCIO-DEMOGRAPHIC INFORMATION OF WOMAN** گھر ۾ رهڻ سهڻ متعلق معلومات			
1	How old are you?<br>توهان جي عمر ڪيتري آهي؟ (پورن سالن ۾)	[ ][ ] Age in Years (پورن سالن ۾)	
2	Religion of the woman عورت جومذهب	1. Muslim مسلمان<br>2. Hindu هندو<br>98. Others (specify) (لکو) يا ٻيو ڪجھ ___________	
3	Mother's Education توهان گهڻا درجا پڑهيل آهيو؟	1. Illiterate (cannot read/write) اڻپڑهيل (لکي يا پڑهي نٿي سگهي)<br>2. Primary complete پنج جماعتون مڪمل<br>3. Primary incomplete پنج جماعتن کان گهٽ<br>4. Middle ڇهين کان اٺين ڪلاس تائين<br>5. Secondary نائون ۽ ڏهون ڪلاس<br>6. Intermediate يارهون ۽ ٻارهون<br>7. Graduation ٻارهين ڪلاس کان مٿي<br>98. Others (specify) يا ٻيو ڪجھ (لکو)	
4	Total number of years schooling completed by you?<br>توهان اسڪول جا ڪيترا ڪلاس پڑهيل آهيو (عورت کان پڇو)؟	|___|___|	
5	Are you working currently for earning?<br>ڇا توهان هن وقت روزگار جي لاءِ ڪو ڪم ڪري رهيا آهيو؟	1. Yes ها<br>2. No نه<br>99. Don't Know خبر ناهي	IF NO SKIP TO Q:8<br>اگر نہ تہ سوال8 پڇو
6	What type of work you do?<br>توهان ڪهڙي قسم جو ڪم ڪريو ٿا؟	1. Day laborer مزدور<br>2. Weaving سيبو سٽو ڪرڻ<br>3. Animal husbandry (for sale) جانور وڪڻڻ<br>دڪاندار<br>4. Shopkeeper<br>5. Technical laborer (electrician, plumber, mechanic, baker)<br>هنري فني مزدور (لائٽ وارو، پاڻي جي لائن ٺاهڻ وارو مستري، بيڪري وارو<br>6. Painter/Artist نقاش<br>7. Construction worker بلڊنگ ٺاهڻ وارو<br>8. Farmer هاري<br>9. Domestic helper گهريلو مددگار<br>10. Businessman ڪاروبار ڪرڻ وارو<br>11. Bus/truck driver وارو بس / ٽرڪ ڊرائيور<br>12. Professional پيشاور<br>13. Security agent گارڊ<br>14. Office worker آفيس ۾ ڪم ڪرڻ وارو<br>15. Factory worker فيڪٽري ۾ ڪم ڪرڻ وارو<br>98. Other→ SPECIFY: _________________<br>يا ٻيو ڪجھ (لکو	

7	What is your monthly income?<br>توهانجي ماهاني آمدن ڪيتري آهي؟	|__|__|__|__| Rs	
8	Have you ever worked in field/farm to support your husband or family?<br>ڇا توهان ڪڏهن ٻني ۾ ڪيو آهي؟	1. Yes ها<br>2. No نه	IF NO SKIP TO Q:11<br>اگر نہ تہ سوال11 پڇو
9	Do you currently work in the field/farm?<br>ڇا توهان هن وقت ٻني ۾ ڪم ڪريو ٿا؟	1. Yes ها<br>2. No نه	
10	Do you work barefoot in the field/farm?<br>ڇا توهان پيرن اگهاڙو ٻني ۾ ڪم ڪندا آهيو ؟	1. Yes ها<br>2. No نه	
11	During your illness who decides for treatment?<br>جڏهن بہ توهان بيمار ٿيندا آهيوتہ توهان جي علاج جو فيصلو ڪير ڪندو آهي؟	1. Woman herself عورت پاڻ<br>2. Husband گهر وارو<br>3. Mother in law سس<br>4. Father in law سوهرو<br>98. Others (specify)(لکو) يا ٻيو ڪجھ _______________	
12	Place of residence رهڻ جي جڳھ	1. Urban شهري<br>2. Rural ٻهراڙي	
13	What kinds of roads are near your house?<br>توهان جي گهر جي چوڌاري ڪهڙا روڊ رستا آهن؟<br>(مناسب جوابن تي ٽڪ جو نشان لڳايو)	1. Paved پڪا روڊ رستا<br>2. Non-paved ڪچا روڊ رستا<br>3. No roads near my house ڪي بہ روڊ رستا ڪونهي	
14	Type of residential house?<br>توهان جوگهر ڪهڙي قسم جو آهي؟(انٽرويو وٺندڙ پاڻ جانچ وٺي)	1. Katcha ڪچو<br>2. Pacca پڪو<br>3. Semi pacca اڌڪچو ۽اڌ پڪو	
15	Which of the following do you live in?<br>توهان هيٺين مان ڪنهن ۾ رهو ٿا(هيٺين جوابن کي پڙهو)	1. Own Dwelling پنهنجو گهر<br><br>2. Family Home خاندان جو گهر<br>3. Rented House ڪرائي جو گهر<br>4. Rented Apartment ڪرائي جو فليٽ<br>5. Rented Room ڪرائي جو ڪمرو<br>98. Others (specify) _____________<br>ٻيو ڪجهه→وضاحت ڪريو	
16	How many people live in your household?<br>گهر ۾ گهڻا ماڻهو رهندا آهن؟	|__|__|	
17	How many rooms in this household are used for sleeping?<br>هن گهر ۾ ڪيترا ڪمرا سمهڻ جي لاءِ استعمال ٿيندا آهن؟	|___|___| rooms used for sleeping<br>سمهڻ جي لاءِ استعمال ٿيندڙ ڪمرن جو تعداد لکو	
18	Type of family System<br>ڪهڙي قسم جو خاندان آهي؟	1. Joint Family system هڪ کان وڌيڪ خاندان گڏ رهي ٿو<br>2. Nuclear Family system<br>هڪ هي خاندان آهي ۽ الڳ رهي ٿو<br>98. Others (specify) _______________<br>يا ٻيو ڪجھ (لکو	
19	Do you have Electricity in your house?<br>ڇا توهان جي گهر ۾ بجلي آهي ؟	1. Yes ها<br>2. No نه	
20	Do you possess any of these assets?	1. Own house توهان جو پنهنجو گهر |___|___| 2. Any other house |___|___|	

	چا توهان جي گهرِ ڏنل لسٽ مان ڪا بہ چيزموجود آهي؟<br>(mention the number in front of each item)	هن گهر کان علاوه ڪو ٻيو بہ گهر<br>3. Any shop ڪو دڪان |___|___| 4. Agricultural land |___|___| ڪا بہ زمين ، جيڪڏهن ها تہ ڪيترا ايڪڙ<br>5. Television ٽي وي |___|___| 6. Refrigerator فريج |___|___| 7. Mobile phone موبائيل فون |___|___| 8. A/C اي سي |___|___| 9. A computer ڪمپيوٽر |___|___|
21	Does any member of household have:<br>چا توهان جي گهر جي ڪنهن فرد وٽ هنن مان ڪجهه آهي؟	1. A bicycle?سائيڪل |___|___| 2. A motorcycle? موٽر سائيڪل / اسڪوٽر |___|___| 3. A car? ڪار |___|___| 4. An animal drawn cart? |___|___| جانور سان هلائڻ واري گاڏي<br>5. A Suzuki سوزوڪي |___|___| 6. A truck ٽرڪ |___|___| 7. A Tractor ٽريڪٽر |___|___| 8. A Hi roof هائي روف |___|___| 9. None of the above هنن مان ڪجهه به ڪونهي
22	Do you possess any of these<br>چا توهان وٽ گهر ۾ ڪو بہ جانور/مال مويشي آهي؟	1. Cow ڳئون |___|___| 2. Buffalo مينهن |___|___| 3. Goatٻڪري |___|___| 4. Bull/Ox ڏڳو |___|___| 5. Sheep رڍ |___|___| 6. Camel اٺ |___|___| 7. Hens/Cocks مرغي/ڪڪڙيون |___|___| 8. None of the aboveهنن مان ڪجهه به ڪونهي
23	What is the principal source of potable water (for drinking) that household members use?<br>توهان جي گهر وارن لاءِ پيئڻ جي پاڻي جو بنيادي ذريعو ڪهڙو آهي؟	1. Faucet inside house (public water supply)<br>گهر جو نلڪو (واٽر سپلائي)<br>2. Public tap عوامي نلڪو<br>3. Other pipe source ٻيو ڪو پائيپ جو ذريعو<br><br>4. Public well سيني ماڻهن جي لاءِ / ڳوٺ جو کوهه<br><br>5. Mechanical pump well<br>موٽر / مشين جي ذريعي پاڻي ڪيڻ وارو کوهه<br>6. River/canal واهه / ندي جو پاڻي<br>7. Bore well within the home<br>زمين جي اندر نلڪو (هئنڊ پمپ)<br>98. Other → SPECIFY: ______________________<br>يا ٻيو ڪجهه (لکو<br>99. DON'T KNOW خبر ناهي
24	Do you do anything to the water to make it safer to drink?<br>چا توهان پيئڻ جي پاڻي کي صاف ڪرڻ جي لاءِ ڪجهه ڪندا آهيو؟	1. Yes ها<br>2. No نه<br><br>IF NO SKIP TO Q.26<br>اگر نہ تہ سوال26 پڇو
25	What do you usually do to make water safer to drink?<br>توهان اڪثر پيئڻ جي پاڻي کي صاف ڪرڻ لاءِ چا ڪندا آهيو؟	1. Boil اُبارِيندا آهيو<br>2. Add bleach/chlorine<br>بليچ / ڪلورين جون گوريون استعمال ڪندا آهيو

		3. Strain through a cloth کپڙي سان ڇاڻيندا آهيو<br>4. Use water filter (ceramic, sand, etc.)<br>پاڻي جو فلٽر استعمال ڪندا آهيو<br>5. Solar disinfection<br>سج جي روشني جي ذريعي پاڻي کي صاف ڪندا آهيو<br>6. Let it stand and settle (with alum)<br>ڦٽڪي جو استعمال ڪري، مٽي کي تري ۾ ويهڻ تائين پاڻي کي ڇڏي ڏيندا آهيو<br>98. Other → SPECIFY:_________________<br>يا ٻيو ڪجھ (لکو<br>99. DON'T KNOW خبر ناهي
26	Main material used for your roof?<br>توهان جي گهر جي ڇت ڪهڙي سامان جي ٺهيل آهي؟	1.Man made (هٿرادي ٺهيل آهي) پاڻ ناهي آهي<br>2.Natural قدرتي<br>98. Other → SPECIFY: ______________________<br>يا ٻيو ڪجھ (لکو<br>99. DON'T KNOW خبر ناهي
27	Main material used for your walls?<br>توهان جي گهر جون ڀتيون ڪهڙي سامان مان ٺهيل آهن؟	1.Man made (هٿرادي ٺهيل آهي) پاڻ ناهي آهي<br>2.Natural قدرتي<br>98. Other → SPECIFY: ______________________<br>يا ٻيو ڪجھ (لکو<br>99. DON'T KNOW خبر ناهي
28	Main material of flooring in your dwelling?<br>توهان جي گهر جو فرش ڪهڙي سامان مان ٺهيل آهي؟	1.Man made (هٿرادي ٺهيل آهي) پاڻ ناهي آهي<br>2.Natural قدرتي<br>98. Other → SPECIFY: ______________________<br>يا ٻيو ڪجھ (لکو<br>99. DON'T KNOW خبر ناهي
29	What do you do with trash made by your household?<br>توهان پنهنجي گهر جي گند ڪچري کي ڪيڏانهن ڪندا آهيو؟	1.Burn ساڙهي ڇڏيندا آهيو<br>2.Throw in yard or street گهر جي گهٽي يا اڱڻ ۾اُڇليندا آهيو<br>3.Trash removal service (Public)<br>ميونسپل ڪميٽي وارا ڪچرو کڻي ويندا آهن<br>4.Trash removal service (Private)<br>پنهنجو ڪو ذاتي صفائي وارو ماڻهو ڪچرو کڻي ويندو آهي.<br>98. Other → SPECIFY: ______________________<br>يا ٻيو ڪجھ (لکو<br>99. DON'T KNOW خبر ناهي
30	What is the main type of toilet used by your family?<br>توهان ۽ توهان جو خاندان ڪهڙي قسم جو باٿروم (ڪاڪوس) استعمال ڪندا آهيو؟	1.Own flush toilet پنهنجو فلش وارو ڪاڪوس/ باٿروم<br>2.Shared flush toilet<br>سيني جي لاءِ گڏيل فلش وارو ڪاڪوس/ باٿروم<br>3.Latrine/Traditional pit toilet<br>ليٽرين / عام سوراخ وارو ڪاڪوس<br>4.No toilet facility

		(ليٽرين) ڪاڪوس جي ڪابه سهولت ڪونهي<br>98. Other → SPECIFY: ______________________<br>يا ٻيو ڪجھ (لکو<br>99. DON'T KNOW خبر ناهي
31	Is there open sewage by your house?<br>ڇا توهان جي گھر جي ويجهو کليل گٽر / نالي آهي؟	1. Yes ها<br>2. No نه
**SECTION B: FERTILITY RELATED FACTORS حمل جي نتيجي جي ٽيبل**		
1	At the time of marriage what was your age?<br>توهانجي شادي جي وقت توهانجي عمر ڪيتري هئي؟	|___|___| Years
2	How long have you been married?<br>شادي ٿيڻ کي هن وقت يا هاڻي ڪيترو عرصو ٿيو آهي؟	|___|___| Years
3	Total number of pregnancies<br>ٽوٽل حملن جو تعداد	|___|___|
4	Total number of alive babies<br>توهان ڪيترا ٻيرا اهڙو ٻار ڄڻيو آهي جيڪو ڄمڻ وقت زنده هو	|___|___|
5	Total number of still births<br>توهان ڪيترا ڀيرا مرده ٻار کي ڄڻيو آهي؟	|___|___|
6	Total number of abortions<br>ٽوٽل ضايع حملن جو تعداد	|___|___|
7	Total number of alive children<br>ٽوٽل زنده ٻارن جو تعداد	|___|___|
8	Total number of alive sons<br>ٽوٽل زنده ڇوڪرن جو تعداد	|___|___|
9	Total number of alive daughters<br>ٽوٽل زنده ڇوڪرين جو تعداد	|___|___|
10	How many sons have died?<br>ڪيترا پُٽ ڄمڻ کان پوءِ مري ويا آهن؟	|___|___|
11	How many daughters have died?<br>ڪيتريون ڌيون ڄمڻ کان پوءِ مري ويون آهن؟	|___|___|
12	Date of last delivery.<br>آخري ڄميل ٻار جي تاريخ	___/___/_____
13	Intention of last delivery<br>آخري حمل ٻار جي نيت مَتعلق پُڇو	1. I wanted to become pregnant this time<br>ڇا توهان حاملا ٿيڻ چاهيو پيا<br>2. I wanted to wait until later<br>ڇا توهان ڪُجھ عرصي کان پوءِ حاملا ٿيڻ چاهيو پيا<br>3. I did not want to become pregnant at all<br>ڇا توهان بُلڪل حاملا ٿيڻ نه پيا چاهيو ؟
14	Age difference between your 2nd last and last baby?<br>آخري ۽ انهن کان پهريان ٻا رن جي عمر فرق ڪيترو آهي؟	|___|___| In months
15	Do you currently breastfeed your baby?<br>ڇا هينئر توهان ٻار کي پنهنجو کير بيا ريو پيا ؟	1. Yes ها<br>2. No نه
16	Are you currently pregnant<br>ڇا توهان حمل سان آهيو؟	1. Yes ها<br>2. No Skip to section C نه
17	If yes, what is gestational age for your current pregnancy<br>جيڪڏهن ها ته توهانجي حمل جا گهٽا هفتا آهن؟	|__|__| weeks

18	Intention of current pregnancy<br>موجوده حمل جي نيت مَتعلق پُڇو	1. I wanted to become pregnant this time<br>ڇا توهان حاملا ٿيڻ چاهيو پيا<br>2. I wanted to wait until later<br>ڇا توهان ڪُجھ عرصي کان پوءِ حاملا ٿيڻ چاهيو پيا<br>3. I did not want to become pregnant at all<br>ڇا توهان بُلڪل حاملا ٿيڻ نه پيا چاهيو ؟
**SECTION C :MENSTRUAL CYCLE AND OTHER MEDICAL CONDITIONS**		
1	How old were you when you started having menstrual periods? Age: _________<br>توهانجي ماهواري ڪهڙي سال جي يمر کان شروع ٿي؟	1a. If you cannot remember your exact age, were you:<br>1. Younger than 10 — 10 سالن کان گهٽ<br>2. 10-12 yrs old — 10-12 سالن جي وچ ۾<br>3. 13-15 yrs old — 13-15 سالن جي وچ ۾<br>4. 16 or older — 16 سال يا ان کان مٿي<br>99. DON'T KNOW — خبر ناهي
2	At present which statement best describes your menstrual cycle?<br>هيٺين مان ڪهڙو جواب توهان لاءِ درست آهي؟	1. I'm still having regular periods: The date of my last period was: ___/___/_____<br>منهنجي ماهواري باقاعده آهي . آخري ماهواري جي تاريخ<br><br>2. My periods are irregular: The date of my last period was: ___/___/_____<br>منهنجي ماهواري باقاعده نا آهي<br>3. I'm pregnant, or my last pregnancy ended within the past 2 months, or I'm breast feeding<br>مان حمل سان آهيان يا منهنجي ويم 2 مهينا پهريان ٿي آهي يا ٻار کي کير پياريان پئي<br>4. I've had chemotherapy which has stopped my periods.<br>منهنجي ماهواري دوائن جي ڪري رڪجي وئي آهي<br>5. I've had radiation therapy which has stopped my periods. منهنجي ماهواري شعائن جي ڪري رڪجي وئي آهي<br>98. Other→ SPECIFY: _________________<br>يا ٻيو ڪجھ (لکو)
3	When you are (were) having regular menstrual cycles, how many days are (were) there between periods? _______ Days between periods.<br>جڏهن توهان کي ماهواري باقائدگي سان اچي ٿي ته ٻن ماهوارين جي وچ ۾ ڪيترا ڏينهن هواندا آهن؟	
4	For how many days do (did) you have your period (days of bleeding)? _______ Days<br>ڪيترن ڏينهن تائين توهان کي ماهواري هلي ٿي؟	
5	Between the ages of 18 and 40, excluding times when you may have been on the pill, pregnant, or nursing, which of the following statements BEST describes your menstrual periods? They are (were)…<br>توهانجي ماهواري کي توهان ڪهڙي نموني بيان ڪري سگهو ٿا؟	1. Nearly always regular<br>تقريبا روزانه/باقائدگي سان ايندي آهي . اندازو لڳائي سگهي ٿو ته تاريخ ڇا هوندي<br>2. Fairly Regular — اندازو نتو ڳڻائي سگهي (لڳ ڀڳ باقائده)<br>3. Irregular — باقائدگي سان نٿي اچي<br>4. Don't Know — خبر ناهي
6	During a typical menstrual period, is your bleeding:<br>جڏهن توهانکي ماهواري ايندي آهي، توهانکي ڪيترا ڏينهن خون جاري ٿيندو آهي؟	1. Heavy — تمام گهڻو خون<br>2. Light — هلڪو خون<br>3. Normal — نارمل

7	Which of the following describes the predominant color of your menstrual blood?<br>توهانجي ماهواري وقت خون جو رنگ ڪهڙو هوندو آهي؟	1. Light Red هلڪو ڳاڙهو<br>2. Purple واڱڑائي<br>3. Brown ناسي<br>4. Dark Red تيز ڳاڙهو<br>5. Black ڪارو<br>6. Bright Red تمام تکو ڳاڙهو
8	Is there clotting with your period?<br>ڇا توهانکي ماهواري ۾ خون جو لوٿڙا جاري ٿيندا آهن؟	1. Yes ها<br>2. No نه
9	Do you or have you ever had any of the following diseases? (mark all that apply)<br>ڇا توهانکي هن وقت يا ڪڏهن به انهن مان ڪا بيماري ٿي آهي؟	1. Malaria مليريا<br>2. Cancer ڪينسر<br>3. Respiratory disease ساهه جي بيماري / دم<br>4. HIV ايڊز<br><br>5. Diabetes شگر<br>6. Anemia رت جي ڪمي<br><br>7. Parasites جراثيمي انفيڪشن<br><br>8. TB ٽي بي<br>9. Cardiovascular disease دل جي بيماري<br><br>10. None ڪا بيماري ناهي<br>98. Other → SPECIFY: ______________________<br>يا ٻيو ڪجهه (لکو
10	Do you take any medication, vitamin, mineral, or other supplement?<br>ڇا توهان ڪا دوا يا گوري وغيره کائيندا آهيو؟	1. Yes ها<br>2. No Skip to Q:14 نه
11	Are these supplements contains iron?<br>ڇا انهن ۾ آئرن (فولاد) موجود هوندو آهي؟	1. Yes ها<br>2. No نه<br>3. Don’t Know
12	Are these supplements contains folic acid?<br>ڇا انهن گورين ۾ فولڪ ائسڊ موجود هوندو آهي؟	1. Yes ها<br>2. No نه<br>3. Don’t Know
13	How often do you take this supplement?<br>توهان اهي گوريون ڪيترا دفعا کيئندا آهيو؟	1. Daily: Mention # of tablets per day |__|__| روزانو<br>2. Weekly: Mention # of tablets per week |__|__| هفتيوار<br>3. Monthly: Mention # of tablets per month |__|__| مهانا<br>98. Other→ SPECIFY: _______________<br>يا ٻيو ڪجهه (لکو
14	Have you ever received any blood transfusion?<br>ڇا توهانکي ڪڏهن خون جون بوتلون چڙهيون آهن؟	1. Yes ها<br>2. No Skip to section D نه
15	How many blood bottles?<br>ڪيتريون خون جون بوتلون؟	|__|__| mention number of blood bottles<br>بوتلن جو تعداد لکو

16	Why did you receive blood transfusion?<br>خون چڙهڻ جي وجه /ڪارڻ ڇا هيو؟	1. During pregnancy حمل جي دوران<br>2. Severe Anemia خون جي ڪمي<br>3. Heavy menstrual cycles زياده ماهواري جي ڪري<br>4. Antepartum hemorrhage حمل دوران خون جاري ٿيڻ<br>5. Post-partum hemorrhage ويم ڪانپوءِ<br>6. At the time of delivery ويم دوران خون جاري ٿيڻ<br>7. Due to C-Section آپريشن جي ڪري (پار جي چمڻ)<br>8. Major surgery وڏو آپريشن<br>98. Other → SPECIFY: ____________________<br>يا ٻيو ڪجھ (لکو
**SECTION D : FAMILY PLANNING**		
1	Have you ever heard about any FP methods?<br>ڇا توهان ڪڏهنFPجي طريقي جي باري ۾ ٻڌو آهي؟	1. Yes ها<br>2. No→ if no skip to next section نه
2	Which FP method do you know?<br>ڪهڙو طريقو؟	1. Female Sterilization فيميل اسٽيلائزيشن<br>2. Male Sterilization ميل اسٽيلائزيشن<br>3. IUCD آئي يو سي ڊي<br>4. Injections انجيڪشن<br>5. Implants امپلانٽ<br>6. Pill پل<br>7. Condom ڪنڊوم<br>8. Standard days method اسٽنڊرڊ ڊيس طريقو<br>9. Lactational amen. method ليڪٽيشنل طريقو<br>10. Rhythm method ردهم طريقو<br>11. Withdrawal ود ڊراول<br>98. Other → SPECIFY: ____________________<br>يا ٻيو ڪجھ (لکو
3	Did you ever use any method to delay or avoid getting pregnant?<br>ڇا ڪڏهن توهان ڪو طريقو استعمال ڪيو آهي؟	1. Yes ها<br>2. No→ if no skip to next section نه<br>1. Female Sterilization فيميل اسٽيلائزيشن<br>2. Male Sterilization ميل اسٽيلائزيشن<br>3. IUCD آئي يو سي ڊي<br>4. Injections انجيڪشن<br>5. Implants امپلانٽ<br>6. Pill پل<br>7. Condom ڪنڊوم<br>8. Standard days method اسٽنڊرڊ ڊيس طريقو<br>9. Lactational amen. method ليڪٽيشنل طريقو<br>10. Rhythm method ردهم طريقو<br>11. Withdrawal ود ڊراول<br>98. Other → SPECIFY: ____________________<br>يا ٻيو ڪجھ (لکو
4	Are you currently using any FP method<br>ڇا توهان ڪو طريقو استعمال ڪريو پيا؟	1. Yes ها<br>2. No نه
5	Which FP method/s are you currently using?<br>ڪهڙو طريقو؟	1. Female Sterilization فيميل اسٽيلائزيشن<br>2. Male Sterilization ميل اسٽيلائزيشن<br>3. IUCD آئي يو سي ڊي<br>4. Injections انجيڪشن

		5. Implants امپلانٽ<br>6. Pill پل<br>7. Condom ڪندوم<br>8. Standard days method اسٽنبرڊ ڊيس طريقو<br>9. Lactational amen. method ليڪٽيشنل طريقو<br>10. Rhythm method ردهم طريقو<br>11. Withdrawal ود ڊراول<br>98. Other → SPECIFY: ____________________<br>يا ٻيو ڪجھ (لکو
6	Since how long you are using current method?<br>ڪيتري وقت کان ؟	|____|months
**SECTION E : NUTRITION AND FOOD**		
1	How many meals per day do you usually eat?<br>اڪثر ڪري توهان ڏينهن ۾ گهڻا ويلا ماني کائو ٿا؟	|____| number of meals per day
2	Do you usually have breakfast?<br>ڇاتوهان عام طور تي ناشتو ڪندا آهيو؟	1. Yes ها<br>2. No (Skip to Q.4) نه<br>99. DON'T KNOW خبر ناهي
3	If yes, how many times did you have breakfast in the last week, that is, in the last 7 days?<br>جيڪڏهن ها ته توهان پنئين هفتي ۾ ڪيترا ڀيرا ناشتو ڪيو؟	1. Every day (seven precious days) روزانه<br>2. 4-6 times per week چار کان ڇهه دفعا<br>3. 1-3 times per week 1- تي دفيا<br>4. Never ڪڏهن به نه
4.	Did you worry that you would not have enough food?<br>ڇا توهان پريشان هئا ته توهان وٽ کاڌي کاڻ جي لاءِ کاڌو پورو ڪونهي؟	1. Yes ها<br>2. No → SKIP TO Q.6 نه
5.	If yes, How often did this happen?<br>جيڪڏهن ها ته، ڪيترا ڀيرا؟	Use the following codes for all frequency of occurrence responses (1a – 9a)<br>a1 کان وني 9a تائين ڏنل جوابن جي لاءِ هيٺ ڏنل ڪوڊ استعمال ڪريو.<br>1 = Rarely (1-2 times in the past four weeks)<br>الاهي گهٽ (پنين چار هفتن ۾ 1 کان 2 ڀيرا)<br>2 = Sometimes (3-10 times in the past four weeks)<br>ڪڏهن ڪڏهن (پنين چار هفتن ۾ 3 کان 10 ڀيرا)<br>3 = Often (>10 times in the past four weeks)<br>اڪثر ڪري (پنين چار هفتن ۾ 10 ڀيرن کان وڌيڪ)
6.	Were you not able to eat the kinds of foods you preferred because of a lack of money or goods for food purchases?<br>ڇا توهان پنهنجو پسنديده کاڌو ان جيڪري ڪو نه کاڌو ڇو جو توهان وٽ پئسا ڪو نه هئا؟	1. Yes ها<br>2. No → SKIP TO Q.8 نه
7.	If yes, How often did this happen?<br>جيڪڏهن ها ته، ائين ڪيترا ڀيرا ٿيو؟	|____|
8.	Did you have to eat a limited variety of foods due to a lack of resources?<br>ڇا توهان کي گهٽ وسيلن هجڻ جي ڪري گهٽ قسمن جا کاڌا کائڻا پيا؟	1. Yes ها<br>2.No → SKIP TO Q.10 نه
9.	If yes, How often did this happen?<br>جيڪڏهن ها ته، ائين ڪيترا ڀيرا ٿيو؟	|____|

10.	Did you have to eat some foods that you really did not want to eat because of a lack of resources to obtain other types of food?<br>["A food you really did not want to eat" might include…]<br>چا توهان کي گهڻ وسيلن جي ڪري نا پسنديده کاڌا کاڌا پيا؟	1. Yes ها<br>2.No → SKIP TO Q.12 نه
11.	If yes, How often did this happen?<br>جيڪڏهن ها ته، ائين ڪيترا پيرا ٿيو؟	|____|
12.	Did you have to eat a smaller meal than you felt you needed because there was not enough food?<br>چا توهان کي گهڻ کاڌو هئڻ ڪري پنهنجي ضرورت جي مطابق گهڻ کاڌو کاڌو پيو؟	1. Yes ها<br>2.No → SKIP TO Q.14 نه
13.	If yes, How often did this happen?<br>جيڪڏهن ها ته، ائين ڪيترا پيرا ٿيو؟	|____|
14.	Did you have to eat fewer meals in a day because there was not enough food?<br>چا گهڻ کاڌو هئڻ جي ڪري توهان هڪ ڏينهن ۾ گهڻ ماني / ويلا کاڌا ؟	1. Yes ها<br>2.No → SKIP TO Q.16 نه
15.	If yes, How often did this happen?<br>جيڪڏهن ها ته، ائين ڪيترا پيرا ٿيو؟	|____|
16.	Was there ever no food to eat of any kind in your household because of a lack of resources to get food?<br>چا ڪڏهن ائين ٿيو جو گهڻ وسيلن هئڻ ڪري گهر ۾ کائڻ لاءِ ڪجهه به نه هجي؟	1. Yes ها<br>2.No → SKIP TO Q.18 نه
17.	If yes, How often did this happen?<br>جيڪڏهن ها ته، ڪيترا پيرا ائين ٿيو؟	|____|
18.	Did you go to sleep at night hungry because there was not enough food?<br>چا ڪڏهن ائين ٿيو کاڌو گهڻ هُجڻ جي ڪري توهان بکايل سمهي رهيا هجو ؟	1. Yes ها<br>2.No → SKIP TO Q.20 نه
19	If yes, How often did this happen?<br>جيڪڏهن ها ته، ائين ڪيترا پيرا ٿيو؟	|____|
20.	Did you go a whole day and night without eating anything because there was not enough food?<br>چا ڪڏهن گهڻ کاڌو هئڻ ڪري توهان سڄو ڏينهن ۽ سڄي رات کاڌي کائڻ جي بغير گذاري ؟	1. Yes ها<br>2.No → SKIP TO Q.22 نه
21.	If yes, How often did this happen?<br>جيڪڏهن ها ته، ائين ڪيترا پيرا ٿيو؟	|____|
22.	SUM OF THE VALUES IN Q.7-Q.21<br>سوال 7 کان وٺي سوال 21 تائين جواب جمع ڪري لکو.	|____|
23.	How many meals per day do you usually eat?<br>اڪثر ڪري توهان ڏينهن ۾ گهڻا ويلا ماني کائو ٿا؟	|____|
24.	Have you received any food assistance in the last 4 weeks?<br>گذريل چار هفتن ۾ چا توهان کاڌي جي لاءِ ڪنهن جي مدد حاصل ڪئي آهي؟	1. Yes ها<br>2.No→ SKIP TO Q.26 نه
25.	Specify who provided assistance:<br>وضاحت ڪريو ڪنهن مدد ڪئي	________________________
26.	Do you drink tea?<br>چا توهان چانئي پيئندا آهيو؟	1. Yes ها<br>2. No (Skip to Q.28) نه

		99. DON’T KNOW خبر ناهي
27.	If yes how many cups you per day?<br>جيڪڏهن ها ته ڏينهن ۾ ڪيترا دفعا؟	1. 1-2 cups 1-2 ڪپ<br>2. 3-4 cups 3-4 ڪپ<br>3. 5-6 cups 5-6 ڪپ<br>4. 6 +
28	Does your house have a garden?<br>چا توهان جي گهر ۾ باغ آهي؟	1. Yes ها<br>2. No (Skip to Q.30) نه
29.	Is food for the family grown in the garden?<br>چا توهان پنهنجي خاندان جي لاءِ ڪاڌو باغ ۾ پوکيندا آهيو؟	1. Yes ها<br>2. No نه
30.	Do you eat vegetables?<br>چا توهان سبزيون کائيندا آهيو؟	1. Yes ها<br>2. No (Skip to Q.32) نه<br>3. Don’t know خبر ناهي
31.	If yes Did you eat:<br>Pieces Portion Never 1/month 2-3/mo 1/wk 2-3/wk 1/day<br>جيڪڏهن ها ته	1. Pumpkin ڪدو<br>2. Carrot گجر<br>3. Spinach پالڪ<br>98. Other → SPECIFY: ____________________<br>يا ٻيو ڪجھ (لکو
32.	Do you eat meat?<br>چا توهان گوشت کائيندا آهيو؟	1. Yes ها<br>2. No نه<br>99. DON’T KNOW خبر ناهي
33.	If yes Do you eat:<br>Pieces Portion Never 1/month 2-3/mo 1/wk 2-3/wk 1/day<br>جيڪڏهن ها ته	1. Mutton ننڍو گوشت<br>2. Beef وڏو گوشت<br>3. Chicken مرغي<br>4. Fish مڇي<br>98. Other → SPECIFY: ____________________<br>يا ٻيو ڪجھ (لکو
**SECTION F. INDOOR AIR POLLUTION AND SUBSTANCE ABUSE**		
1	Where is the cooking usually done?<br>توهان عام طور تي ڪاڌو ڪٿي پچائيندا آهيو؟	1. In the house گهر جي اندر<br>2. In a separate building الڳ عمارت ۾<br>3. Outdoors گهر کان ٻاهر<br>98. Other → SPECIFY: ____________________<br>يا ٻيو ڪجھ (لکو
2	What type of fuel does your household mainly use for cooking?<br>توهان پنهنجي گهر ۾ رڌ پچاءُ جي لاءِ ڪهڙو ٻارڻ استعمال ڪندا آهيو؟	1. Electricity بجلي<br>2. LPG ايل پي جي (سلينڊر واري گئس)<br>3. Natural Gas قدرتي گئس<br>4. Kerosene بائيو گئس<br>5. Coal ڪوئلو<br>6. Charcoal چارڪول<br>7. Wood ڪاٺيون

		8. Straw/Shrubs/Grass گاهه گند<br>9. Animal Dung جانور جا چيٹا<br>10. No food cooked in household<br>گهر ۾ کاڌو ڪو نه پچندو آهي<br>98. Other → SPECIFY: ____________________<br>يا ٻيو ڪجھ (لکو
3.	Does your baby sleep or play in the same room where you cook?<br>چا توهان جو ٻار ساڳئي ڪمري ۾ اندر رهندو آهي يا سمهندو آهي جتي توهان رڌ پچاءُ ڪندا آهيو؟	1. Yes ها<br>2. No نه<br>3. N/A (no baby) لاڳو ڪونهي (ٻار ڪونهي)
4.	Do you have a separate room which is used as a kitchen?<br>چا توهان وٹ ڪو الڳ ڪمرو آهي جيڪو رڌ پچاءُ جي لاءِ الڳ سان استعمال ٿينبدو آهي؟	1. Yes ها<br>2. No نه
5.	Does this room have a chimney for smoke removal?<br>چا هن ڪمري مان دونهن نڪرڻ جو رستو / چمني آهي؟	1. Yes ها<br>2. No نه
6.	Does anyone smoke inside your house?<br>توهان جي گهر جو ڪو به فرد ڪيترا ڀيرا تمباڪو نوشي ڪندو / ڪندي آهي؟	1. Yes ها<br>2. No (Skip to Q.8) نه
7.	How often does anyone smoke inside your house?<br>توهان جي گهر جو ڪو به فرد ڪيترا ڀيرا تمباڪو نوشي ڪندو / ڪندي آهي؟	1. Daily هر روز<br>2. Weekly هر هفتي<br>3. Monthly هر مهيني<br>4. <Monthly مهيني کان گهٽ<br>5. Never ڪڏهن به نه
8.	Do you currently smoke tobacco, use hukka or history of Pica?<br>چا توهان هيئر تمباڪو نوشي ڪندا آهيو؟	1. Yes ها<br>2. No (Skip to Q.9) نه
8.1	On average, how many cigarettes per day?<br>سراسري روز ڪيترا سگريٽ پيئندا آهيو؟	|__|__|
8.1a	Since how long you are smoking?<br>توهان ڪڏهن کان سگريٽ پيو ٿا؟	|__|__|-|__|__| year- months
8.2	If use Hukka how many time per day?<br>جيڪڏهن هڪو ته ڏينهن ۾ گهڻا دفعا؟	|__|__|
8.2a	Since how long you are using hukka?<br>توهان ڪڏهن کان هڪو پيو ٿا؟	|__|__|-|__|__| year- months
8.3	If history of pica, which type?<br>ڪهڙي قسم جو مثلا؛ مٽي، ميٽ وغيره	1.Dirt مٽي<br>2. Paint رنگ<br>3.Ice برف<br>4. Bleaching clay ميٽ ئ ملتاني مٽي<br>98. Other → SPECIFY: ____________________<br>يا ٻيو ڪجھ (لکو
8.3a	How many times you use the identified item per day?<br>ڏينهن ۾ ڪيترا ڀيرا؟	|__|__|
9	Do you currently chew tobacco?<br>چا توهان کو تمباڪو ، پان، گڏڪو کائيندا آهيو؟	1. Yes ها<br>2. No نه

10	What type of tobacco do you chew?<br>ڪيتري وقت کان؟	1. Gutka<br>2. Pan<br>3. Mawa<br>4. Tambako<br>98. Other → SPECIFY: ______________________<br>يا ٻيو ڪجھ (لکو
11	Since how long you are using this form of Tabacco?<br>ڪيتري وقت کان؟	|___|___|-|___|___| year- months
**SECTION G. MATERNAL ANTHROPOMETRY**		
1	Record height<br>ماءُ جي قد جي ماپ لکو	|___|___|___|.|___| cm
2	Record weight<br>ماءُ جو وزن لکو	|___|___|___|.|___| cm
3	Calculate and record BMI<br>ماءُ جوBMIماپي لکو	|___|___|.|___| kg/m2
4	Record head circumference<br>ماءُ جي مٿي جي گولائي جي ماپ لکو	|___|___|___|.|___| cm
5	Record MUAC<br>ماءُ جي مٿئين ٻانهن جي وچ واري حصي جي گولائي ماپي لکو	|___|___|.|___| cm
6	Record waist circumference<br>ماءُ جي چيلهه جي گولائي جي ماپ لکو	|___|___|___|.|___| cm
7	Record hip circumference<br>ماءُ جي ڏڏن جي گولائي جي ماپ لکو	|___|___|___|.|___| cm
**SECTION H. Lab Reports**		
1	CBC Report	
2	HB (using heamacue)	|___|___|.|___| g/dL
3	Serum Folic Acid	
4	Serum B12 Levels	
5	Serum Iron Levels	
6	Serum Feritin Levels	
7	HB Electrophoresis Report	
8	Malaria ICT	
9	Stool DR	
**SECTION I. Husband Information** (Questions to be asked from Husband)		
1	What is your age?<br>توهانجي عمر ڪيتري آهي؟	|__|__| in years

2	Husband's Education.<br>توهانجي ڪيتري تعليم حاصل ڪئي آهي؟	1. Illiterate (cannot read/write)<br>اڻپڙهيل(لکي يا پڙهي نٿو سگهي<br>2. Primary complete پنج جماعتون مڪمل<br>3. Primary incomplete پنج جماعتن کان گهٽ<br>4. Middle ڇهين کان اٺين ڪلاس تائين<br>5. Secondary نائون ۽ ڏهون ڪلاس<br>6. Intermediate يارهون ۽ ٻارهون<br>7. Graduation ٻارهين ڪلاس کان مٿي<br>98. Other → SPECIFY: ____________________<br>يا ٻيو ڪجھ (لکو
3	Total number of years schooling completed by you?<br>توهان اسڪول جا ڪيترا ڪلاس پڙهيل آهيو (عورت کان پڇو)؟	|___|___|
4	Do your work?<br>ڇا توهان جو مڙس ڪو ڪم ڪندو آهي؟	1. Yes ها<br>2. No If No skip to Q:7 نه<br>99. DON'T KNOW خبر ناهي
5	What is your occupation?<br>هو ڪهڙو ڪم /ڌنڌو ڪندو آهي ؟	1. Day laborer مزدور<br>2. Weaving سيبو سٽو ڪرڻ<br>3. Animal husbandry (for sale) جانور وڪڻ<br>دڪاندار<br>4. Shopkeeper<br>5. Technical laborer (electrician, plumber, mechanic, baker)<br>هنري فني مزدور (لائٽ وارو، پاڻي جي لائن ناهڻ وارو مستري، بيڪري وارو<br>6. Painter/Artist نقاش<br>7. Construction worker بلڊنگ ناهڻ وارو<br>8. Farmer هاري<br>9. Domestic helper گهريلو مددگار<br>10. Businessman ڪاروبار ڪرڻ وارو<br>11. Bus/truck driver وارو بس / ٽرڪ ڊرائيور<br>12. Professional پيشاور<br>13. Security agent گارڊ<br>14. Office worker آفيس ۾ ڪم ڪرڻ وارو<br>15. Factory worker فيڪٽري ۾ ڪم ڪرڻ وارو<br>98. Other→ SPECIFY: __________________<br>يا ٻيو ڪجھ (لکو

6	What is your monthly income?<br>ماهانا آمدن ڪيتري آهي؟	|___|___|___|___| RS
7	Have you ever worked in field/farm to support your husband or family?<br>ڇا توهان ڪڏهن ٻني ۾ ڪيو آهي؟	1. Yes ها<br>2. No IF NO SKIP TO Q:9 نه
8	Do you currently work in the field/farm?<br>ڇا توهان هن وقت ٻهنئي ۾ ڪم ڪريو ٿا؟	1. Yes ها<br>2. No نه
9	Do you work barefoot in the field/farm?<br>ڇا توهان پيرا ڳهاڙو ٻني ۾ ڪم ڪندا آهيو ؟	1. Yes ها<br>2. No نه
10	Do you currently smoke tobacco?<br>ڇا توهان سگريٽ پيئندا آهيو؟	1. Yes ها<br>2. No IF NO SKIP TO Q:13 نه
11	On average how many cigarettes per day?<br>هڪڙي ڏيهن ۾ گهڻ؟	|__|__|
12	Since how long you are smoking?<br>توهان ڪڏهن کان سگريٽ پيو ٿا؟	|__|__|-|__|__| year- months
13	Do you currently chew tobacco?<br>ڇا توهان کو تمباڪو ، پان، گڏڪو کائيندا آهيو؟	1. Yes ها<br>2. No نه
14	What type of tobacco do you chew?<br>ڪيتري وقت کان؟	1. Gutka گٽکا<br>2. Pan پان<br>3. Mawa ماوا<br>4. Tambako تمباکو<br>98. Other → SPECIFY: _____________________<br>يا ٻيو ڪجھ (لکو
15	Since how long you are using this form of Tabacco?<br>ڪيتري وقت کان؟	|__|__|
16	Do you drink alcohol?<br>ڇا توهان شراب پئندا آهيو؟	1. Yes ها<br>2. No نه
17	How frequently drink alcohol?<br>توهان ڪيترا دفعا شراب پيو ٿا؟	1. Daily روزانو<br>2. Weekly هفتي ۾<br>3. Monthly مهيني ۾<br>4. Never ڪڏهن به نه
18	Is your wife your relative?<br>ڇا توهانجي ذال تومانجي مائٽن مان آهي؟	1. Yes ها<br>2. No نه
19	What relationship you had?<br>ڪهڙي مائٽن مان	a. First cousin<br>b. Second cousin<br>c. Distant relative<br>d. No relationship<br>98. Other → SPECIFY: _____________________<br>يا ٻيو ڪجھ (لکو
